# A changing profile of infective endocarditis at a tertiary hospital in China: a retrospective study from 2001 to 2018

**DOI:** 10.1186/s12879-019-4609-8

**Published:** 2019-11-08

**Authors:** Zuning Ren, Xichao Mo, Hongjie Chen, Jie Peng

**Affiliations:** Department of Infectious Diseases, Nanfang Hospital, Southern Medical University, Guangzhou, 510515 China

**Keywords:** Infective endocarditis, Clinical characteristics, Risk factors, Mortality, Retrospective study, Independent predictors

## Abstract

**Background:**

Infective endocarditis (IE) is a lethal disease which has been changing significantly over the past decades; however, information about IE in China remains scarce. This study surveyed the changes in clinical characteristics of IE at a tertiary hospital in south China over a period of nearly 18 years.

**Methods:**

Medical records with IE patients consecutively hospitalized between June 2001 and June 2018 were selected from the electronic medical records system in Nanfang Hospital of Southern Medical University. Data were divided by admission time into two groups equally: early-period group, June 2001 to December 2009 and later-period group, January 2010 to July 2018.

**Results:**

A Total of 313 IE patients were included in our study. Compared with the early-period group, patients in the later-period group included fewer intravenous drug users (IVDUs), older age at onset, reduced development of pulmonary embolism, less renal dysfunction, decreased proportion of *Staphylococcus aureus* infection and fewer vegetations observed in the right heart by echocardiography. The later-period group also showed a higher proportion of ischemic strokes and higher proportion of positive microbiological findings compared with the early-period group. The in-hospital mortality remained about the same between the two periods and the multivariate analysis identified intravenous drug addicted, prosthetic valve endocarditis, hemorrhagic stroke, acute congestive heart failure, renal insufficiency, left-sided endocarditis, early surgical as independent predictors of in-hospital mortality.

**Conclusions:**

Our study demonstrated a dramatic change in the profile of IE over a period of 18 years at a tertiary hospital in south China and presented several independent predictors of in-hospital mortality. The geographic variations observed in our study will be of important value to profile the clinical feature of China and offer the reference for clinical decisions in our region.

## Background

Infective endocarditis is a lethal disease caused by various pathogens such as bacteria, fungi, and rickettsia that directly invade the cardiac valves or mural endocardium [1]. The profile of IE has been changing significantly over the past decades [2]. Overall, IE related to rheumatic diseases has dramatically decreased in developed countries, being gradually replaced by IE associated with congenital heart disease, degenerative heart valve disease, prosthetic valves and cardiac implantable electronic devices [3]. Staphylococci, which are most often related to healthcare and invasive procedures, have overtaken streptococci as the most common pathogen of IE [4]. The average age of patients has also been increasing [5]. In contrast, rheumatic disease remains a key predisposing factor in developing countries, and streptococci are still the most common cause of IE. In countries with reduced IVDU, right heart IE has also decreased; but in some regions such as eastern Europe, IVDU remains a problem and right-sided IE continues to occur [6]. Many developed countries have a wealth of prospective or retrospective studies for IE [7–9]. However, there have been few studies of IE in China compared to other countries [10]. To better profile the features of IE and the changes in clinical characteristics at Nanfang Hospital, a tertiary hospital in southern China, and find out the independent predictors of in-hospital mortality, we collected and analyzed the data from consecutive 313 cases of IE over a period of 18 year.

## Methods

### Diagnostic criteria

The definition of cases was based on the European Society of Cardiology (ESC) algorithm for diagnosis of infective endocarditis (2015 edition) [11], which mainly includes the pathological diagnostic criteria and the modified Duke criteria.

Pathological examination served as the gold standard for diagnosing IE, which must meet at least one of the following criteria: microorganisms demonstrated by culture or on histological examination of a vegetation, a vegetation that has embolized, or an intracardiac abscess specimen or the presence of pathological lesions, vegetation or intracardiac abscesses by histological examination showing active endocarditis.

The modified Duke criteria (adapted from Li et al. [12]) were used for clinical diagnosis with cases classified as either definite or suspected. For a diagnosis of definite IE, the patient must meet two major criteria, or one major criterion and three minor criteria, or five minor criteria. For a diagnosis of suspected IE, the patient must meet one major criterion and one minor criterion or three minor criteria. Major criteria include: (1) blood cultures positive for typical microorganisms consistent with IE from two separate blood cultures, microorganisms consistent with IE from persistently positive blood cultures; (2) imaging positive for IE by transthoracic echocardiogram; and (3) definite paravalvular lesions by cardiac CT. Minor criteria include: (1) predisposing heart condition or injection drug use; (2) fever of > 38 °C; (3) vascular phenomena including those detected by imaging only, major arterial emboli, septic pulmonary infarcts, infectious mycotic aneurysm, intracranial hemorrhage, conjunctival hemorrhages, and Janeway’s lesions; (4) immunological phenomena, glomerulonephritis, Osler’s nodes, Roth’s spots, or rheumatoid factor; (5) microbiological evidence, positive blood culture, but does not meet a major criterion as noted above or serological evidence of active infection with organisms consistent with IE. To exclude misdiagnosed cases as sepsis, non-infective endocarditis and rheumatic myocarditis, suspected patients should either show intracardiac vegetations by echocardiography with an evidence of bacterial infection, or meet the pathological diagnostic criteria.

Health care-associated IE was considered likely if any of the following had occurred: the patient had received intravenous therapy at home, received wound care or specialized nursing care through a health care agency, family, or friends, self-administered intravenous medical therapy in the past 30days, was examined at a hospital or hemodialysis clinic or received intravenous chemotherapy in the past 30 days, was hospitalized in an acute care hospital for two or more days in the previous 90 days before the infection, or resided in a nursing home or long-term care facility [13].

Early surgery was defined as surgery within 20 days after diagnosis of IE [14]. On the contrary, late surgery was defined as surgical intervention beyond 20 days after diagnosis.

In-hospital mortality was defined as death from any cause during hospitalization.

### Study sample

Nanfang Hospital of Southern Medical University is a large tertiary teaching comprehensive hospital at Guangdong province in southern China with in-patient quantity up to 119,000 statistically in 2018, where surgery quantity in cardiothoracic surgery surpasses 1000 per year. We consecutively collected 313 inpatients diagnosed with IE through the electronic medical records system of Nanfang Hospital between June 2001 and June 2018. They were divided into two groups according to their time of admission: early-period group, from June 2001 to December 2009, and later-period group, from January 2010 to July 2018.

This clinical study was a retrospective and descriptive study consistent with the principles of the Helsinki declaration.

Data included demographic information, predisposing factors, clinical manifestations, laboratory tests including blood work and biochemical measurements, echocardiography results, causative microorganisms, pathologic findings and therapeutic outcomes. The outcomes included improvement at discharge of clinical symptoms, normal laboratory indicators, negative blood cultures and echocardiograms and worsening at discharge with abandonment of treatment because of poor efficacy or death.

### Statistical method

All analyses were performed using SPSS version 25.0.0. Continuous variables fitting a normal distribution were expressed as mean ± standard deviation. Categorical variables were expressed as frequency and percentage. Univariate comparisons were evaluated with the use of the independent sample *t* test for continuous variables, and Chi-squared tests or Fisher’s exact test for categorical variables, if appropriate. Variables with theoretical clinical importance and those that achieved a *P* value of < 0.10 in the univariate analysis were included in the binary logistic regression analysis. A forward conditional method was used to select the most useful predictors of the in-hospital mortality. A value of *P* <  0.05 was considered significant.

## Results

### Basic information

A total of 313 IE patients were consecutively collected in this study, with 97 patients enrolled in the early-period group and 216 patients in the later-period group. Table 1 shows the basic information of the 313 patients. The later-period group was on average older (44.9 ± 15.4 yrs. vs 36.5 ± 15.2 yrs., *P* <  0.001, 4.692–12.064), mainly due to more patients aged 41–60 years old (43.1% vs. 23.7%, OR = 2.433, CI: 1.418–4.174), and fewer patients aged 21–40 (35.2% vs. 56.7%, OR = 0.415, CI: 0.254–0.676). Each group had a similar male-female ratio, approximately 2.6:1 (72.2%). The top five departments that IE patients were initially admitted were cardiology (28.4%), cardiothoracic surgery (25.6%), infectious disease (15.0%), respiratory (7.7%), and nephrology (5.1%). The proportion of patients in the respiratory department declined in the later-period group (4.6% vs 14.4%, OR = 0.288, CI: 0.123–0.674). Regarding the factors predisposing to IE, 21 cases (6.7%) were considered as healthcare-associated IE. Basic heart diseases were the dominant predisposing factors (45.4%), including rheumatic heart disease (19.2%), congenital heart disease (16.6%) and degenerative heart valve disease (7.7%). IVDUs in the later-period group sharply decreased compared to the early-period group (12.0% vs 25.8%, OR = 0.394, CI: 0.214–0.727). The proportion of diabetic patients was higher in the later-period group, but without statistical significance (10.6% vs. 5.2%).

**Table 1:** Patient characteristics

Variable	Total	Early-period Group	Later-period Group	OR	95% CI		*P*
	*N* = 313	*N* = 97	*N* = 216		Lower	Upper	
Age (year)	42.3 ±15.8	36.5 ± 15.2	44.9 ± 15.4		−4.692	−12.064	< 0.001*
≤ 20	18 (5.8 )	9 (9.3)	ik9 (4.2)	0.425	0.163	1.107	0.072
21–40	131 (41.9)	55 (56.7 )	76 (35.2)	0.415	0.254	0.676	< 0.001
41–60	116 (37.1)	23 (23.7)	93 (43.1)	2.433	1.418	4.174	0.001
≥ 61	48 (15.3)	10 (10.3)	38 (17.6)	1.857	0.884	3.902	0.098
Male	226 (72.2)	70 (72.2	156 (72.2)	1.003	0.588	1.712	0.992
Admission departments							
Department of Cardiology	89 (28.4)	26 (26.8)	63 (29.2)	1.124	0.657	1.923	0.668
Department of Cardiothoracic Surgery	80 (25.6)	19 (19.6)	61 (28.2)	1.616	0.902	2.892	0.105
Department of Infectious Disease	47 (15.0)	10 (10.3)	37 (17.1)	1.798	0.855	3.784	0.118
Department of Respiratory	24 (7.7)	14 (14.4)	10 (4.6)	0.288	0.123	0.674	0.003
Department of Nephrology	16 (5.1)	8 (8.2)	8 (3.7)	0.428	0.156	1.176	0.158
Predisposing factors							
Health care-related	21 (6.7)	3 (3.1)	18 (8.3)	2.848	0.819	9.909	0.087
Basic heart disease	142 (45.4)	47 (48.5)	95 (44.0)	0.835	0.517	1.350	0.462
Congenital heart disease	52 (16.6)	20 (20.6)	32 (14.8)	0.670	0.361	1.243	0.202
Rheumatic heart disease	60 (19.2)	22 (22.7)	38 (17.6)	0.728	0.403	1.313	0.290
Degenerative heart valve disease	24 (7.7)	4 (4.1)	20 (9.3)	2.372	0.789	7.138	0.114
Intravenous drug users	51 (16.3)	25 (25.8)	26 (12.0)	0.394	0.214	0.727	0.002
Prosthetic valve replacement	11 (3.5)	3 (3.1)	8 (3.7)	1.205	0.313	4.644	0.952
Pacemaker	3 (1.0)	1 (1.0)	2 (0.9)	1.000	0.080	10.014	0.897†
Previous IE history	8 (2.6)	2 (2.1)	6 (2.8)	1.357	0.239	6.848	0.987
Recent skin infection	10 (3.2)	3 (3.1)	7 (3.2)	1.049	0.266	4.147	0.781
Diabetes	28 (8.9)	5 (5.2)	23 (10.6)	2.193	0.808	5.951	0.115

Age is presented as mean ± standard deviation. Other variables are presented as count (%). *P* value were estimated by *independent sample t test, Chi-squared tests or †Fisher exact tests. One patient could have two or more underlying predisposing factors

### Manifestations and complications

Table 2 details the manifestations and complications of the 313 IE patients in this study. Our results showed that two groups had similar clinical features, including fever, heart murmurs, hypoproteinemia, anemia, chest pain, heart insufficiency, embolism and hemorrhagic stroke despite radiographically visible splenomegaly (26.4% vs 15.5%, OR = 1.960, CI: 1.046–3.673) and ischemic stroke (27.3% vs 10.3%, OR = 3.269, CI: 1.592–6.714), which was more frequently found in later-period group, while pulmonary embolism (1.9% vs 7.2%, OR = 0.243, CI: 0.069–0.849) and renal failure (6.0% vs 15.5%, OR = 0.350, CI: 0.160–0.768) seemed to appear less often in later-period group.

**Table 2:** Manifestations and complications of 313 patients

Variable	Total	Early-period Group	Later-period Group	OR	95% CI		*P*
	*N* = 313	*N* = 97	*N* = 216		Lower	Upper	
Manifestations							
Fever	262 (83.7)	81 (83.5 )	181(83.8)	1.022	0.535	1.951	0.949
Cardiac murmurs	262 (83.7)	87 (89.7)	175(81.0)	0.491	0.235	1.026	0.055
Splenomegaly	72 (23.0)	15 (15.5)	57 (26.4)	1.960	1.046	3.673	0.034
Chest pain	38 (12.1)	14 (14.4)	24(11.1)	0.741	0.365	1.504	0.405
Janeway lesion	11 (3.5)	6 (6.2 )	5 (2.3)	0.359	0.107	1.208	0.165
Osler nodes	5 (1.6 )	3 (3.1)	2 (0.9)	0.293	0.048	1.781	0.354
Labotory foundings							
Leukocytosis or neutrophilia	199 (63.6)	64 (66.0)	135 (62.5)	0.859	0.520	1.420	0.554
Anemia	247 (78.9)	80 (82.5)	167 (77.3)	0.724	0.392	1.336	0.301
Hypoproteinemia	293 (93.6)	90 (92.8)	203 (94.0 )	1.215	0.469	3.146	0.689
Comlications							
Heart insufficiency	182 (58.1)	60 (61.9)	122 (56.5)	0.800	0.490	1.307	0.343
Acute congestive heart failure	67 (21.4)	23 (23.7)	44 (20.4)	0.823	0.464	1.460	0.505
Embolism	82 (26.2)	19 (19.6)	63 ( 29.2 )	1.690	0.946	3.022	0.075
Ischemic stroke	69 ( 22.0 )	10 ( 10.3 )	59 ( 27.3 )	3.269	1.592	6.714	<0.001
Pulmonary embolism	11 ( 3.5 )	7 ( 7.2 )	4 ( 1.9 )	0.243	0.069	0.849	0.040
Renal infarction	7 (2.2)	3 (3.1)	4 (1.9)	0.591	0.130	2.694	0.785
Splenic infarction	20 (6.4)	4 (4.1)	16 (7.4)	1.860	0.605	5.717	0.272
Hemorrhagic stroke	27 (8.6)	7 (7.2)	20 (9.3)	1.312	0.535	3.215	0.552
Metastatic abscess	18 (5.8)	6 (6.2)	12 ( 5.6 )	0.897	0.326	2.463	0.832
Pulmonary abscess	14 ( 4.5 )	4 ( 4.1 )	10 ( 4.6 )	1.129	0.345	3.692	0.924
Cerebral abscess	7 ( 2.2 )	3 ( 3.1 )	4 ( 1.9 )	0.591	0.130	2.694	0.785
Renal insufficiency	28 ( 8.9 )	15 ( 15.5 )	13 ( 6.0 )	0.350	0.160	0.768	0.007
No complications	27 ( 8.6 )	9 ( 9.3 )	18 ( 8.3 )	0.889	0.384	2.056	0.783

Variables are presented as count (%). *P* value were estimated by Chi-squared tests. One patient could have two or more manifestations and complications

### Blood culture

All 311 IE patients in our study were subjected to blood culture, while blood culture-negative IE (BCNE) patients accounted for 41.8%. The BCNE rate of the later-period group was lower than that of the earlier group (37.0% vs 52.6%, OR = 0.529, CI: 0.325–0.863). The types of microorganism found in the 181 patients with positive blood culture results are summarized in Table 3. Gram-positive cocci (89.0%) dominated the list, followed by Gram-negative bacilli (6.1%), other bacterial (3.9%) and fungi (3.3%). *Staphylococcus aureus* and Streptococcus were separately the most frequent microorganism in earlier-period group and later-period group. The presence of *Staphylococcus aureus* in the later-period group was less common than in the early-period group (20.0% vs 41.3%, OR = 0.355, CI: 0.172–0.732). Instead, with the exception of *Staphylococcus aureus* and streptococcus, other gram-positive cocci, such as Enterococcus (9.6% vs 2.2%) and *Globicatella Sanguis* (6.7% vs 4.3%)*,* got a notably increase (27.4% vs 13.0%, OR = 2.517, CI: 0.985–6.429) in later-period group.

**Table 3:** Microorganism found in the 181 patients with positive blood culture results

Variable	Total	Early-period Group	Later-period Group	OR	95% CI		*P*
	*N* = 181	*N* = 46	*N* = 135		Lower	Upper	
Gram-positive coccus	161 (89.0)	40 (87.0)	121 (89.6)	1.296	0.467	3.599	0.617
*Staphylococcus aureus*	46 (25.4)	19 (41.3)	27 (20.0)	0.355	0.172	0.732	0.004
Streptococcus	76 (42.0)	17 (37.0)	59 (43.7)	1.324	0.665	2.636	0.423
Other	43 (23.8)	6 (13.0)	37 (27.4)	2.517	0.985	6.429	0.048
Enterococcus	14 (7.7)	1 (2.2)	13 (9.6)	4.795	0.610	37.715	0.188
*Globicatella Sanguis*	11 (6.1)	2 (4.3)	9 (6.7)	1.571	0.327	7.554	0.833
Gram-negative bacilli	11 (6.1)	1 (2.2)	10 (7.4)	3.600	0.448	28.922	0.355
Other bacterial	7 (3.9)	2 (4.3)	5 (3.7)	0.846	0.158	4.518	0.805
Fungi	6 (3.3)	3 (6.5)	3 (2.2)	0.326	0.063	1.674	0.352

Variables are presented as count (%). P value were estimated by Chi-squared tests. One patient could be isolated two or more kinds of causative microorganisms from blood culture

### Echocardiography

All patients underwent a transthoracic echocardiography (TTE) examination and 274 (87.5%) showed positive results (Table 4). Only 3 patients of prosthetic valve endocarditis, with negative TTE results, were confirmed by transesophageal echocardiography (TOE). The proportion of negative TTE results was 45.5 and 11.3% respectively for prosthetic valve endocarditis and native valve endocarditis. There were significantly more negative results in the later-period group than in the early-period group (15.3% vs 6.2%, OR = 2.735, CI: 1.106–6.764). We observed that 199 (63.6%) cases were left-sided endocarditis, 60 (19.2%) cases were right-sided endocarditis, 9 (2.9%) cases showed vegetations on both sides of cardiac valve, and 6 cases developed vegetations on non-valvular endocardium. The later-period group presented a lower proportion of right-sided endocarditis compared to the early-period group (16.2% vs 25.8%, OR = 0.557, CI: 0.311–0.996), especially for endocarditis on tricuspid valve (14.8% vs 24.7%, OR = 0.529, CI: 0.292–0.959).

**Table 4:** Echocardiography results of 313 patients

Variable	Total	Early-period Group	Later-period Group	OR	95% CI		*P*
	N = 313	N = 97	N = 216		Lower	Upper	
Vegetation							
No vegetation	39 (12.5)	6 (6.2)	33 (15.3)	2.735	1.106	6.764	0.024
Left cardiac valve	199 (63.6)	60 (61.9)	139 (64.4)	1.113	0.678	1.827	0.671
Mitral valve	105 (33.5)	30 (30.9)	75 (34.7)	1.188	0.711	1.986	0.511
Aortic valve	72 (23.0)	23 (23.7)	49 (22.7)	0.944	0.536	1.663	0.842
Mitral and aortic valve	22 (7.0)	7 (7.2)	15 (6.9)	0.959	0.378	2.434	0.931
Right cardiac valve	60 (19.2)	25 (25.8)	35 (16.2)	0.557	0.311	0.996	0.047
Tricuspid valve	56 (17.9)	24 (24.7)	32 (14.8)	0.529	0.292	0.959	0.034
Pulmonary valve	4 (1.3)	1 (1.0)	3 (1.4)	1.352	0.139	13.166	0.777
Both left and right cardiac valve	9 (2.9)	2 (2.1)	7 (3.2)	1.591	0.324	7.802	0.833
Peripheral abscess	14 (4.5)	5 (5.2)	9 (4.2)	0.800	0.261	2.453	0.924
Severe regurgitation	190 (60.7)	57 (58.8)	133 (61.6)	1.124	0.690	1.833	0.638

Variables are presented as count (%). *P* value were estimated by Chi-squared tests

### Outcomes and predictors of in-hospital mortality

All patients received antibiotic therapy, with 160 (51.1%) submitted to early surgery. Twenty-seven cases (8.6%) were submitted to late surgical intervention based on their specific condition such as hemodynamic instability, uncontrolled sepsis, shock and organ failure (Table 5).

**Table 5:** Treatment regimen and outcomes of 313 IE patients

Variable	Total	Early-period Group	Later-period Group	OR	95%CI		*P*
	N = 313	N = 97	N = 216		lower	upper	
Treatment regimen							
Antibiotic plus surgery	187 (59.7)	57 (58.8)	130 (60.2)	1.061	0.652	1.727	0.812
Early surgery	160 (51.1)	48 (49.5)	112 (51.9)	1.099	0.681	1.775	0.698
Late surgery	27 (8.6)	9 (9.3)	18 (8.3)	0.889	0.384	2.056	0.783
Death	35 (11.2)	13 (13.4)	22 (10.2)	0.733	0.352	1.523	0.404
Acute heart failure	14 (4.5)	4 (4.1)	10 (4.6)	1.129	0.345	3.692	0.924
Cerebrovascular events	10 (3.2)	3 (3.1)	7 (3.2)	1.049	0.266	4.147	0.781
Septic shock and multiple organ failure	9 (2.9)	5 (5.2)	4 (1.9)	0.347	0.091	1.322	0.211
Others	2 (0.6)	1 (1.0)	1 (0.5)	0.447	0.028	7.213†	0.524†

Variables are presented as count (%). *P* value were estimated by Chi-squared tests or †Fisher exact tests

A total of 35 patients (11.2%) died in hospital, 11 in the early-period group and 24 in the later-period group. Of these, 14 died from acute heart failure, 10 from cerebrovascular events, 9 from septic shock and multiple organ failure, and 1 each from severe arrhythmia and acute myelitis. There was no significant difference in in-hospital mortality between two groups.

Multivariate analysis of the clinical variables found to have statistical significance in the univariate analysis (Table 6) identified the following as independent predictors of in-hospital mortality: intravenous drug addicted (OR = 4.290, CI: 1.098–16.758), prosthetic valve endocarditis (OR = 7.374, CI: 1.177–46.179), hemorrhagic stroke (OR = 5.804, CI: 1.830–18.413), acute congestive heart failure (OR = 10.607, CI: 3.842–29.284), renal insufficiency (OR = 9.268 CI: 2.924–29.382), left-sided endocarditis (OR = 5.606, CI:1.461–21.512), and early surgery (OR = 0.099, CI:0.030–0.330) (Table 7). The goodness-of-fit of the multivariable model was determined by Hosmer–Lemeshow test (Chi-square = 1.562, *P* = 0.955).

**Table 6:** Factors associated with in-hospital mortality: univariate analysis

Factor	Category	Number	Deaths	OR	95% CI		*P*
					lower	upper	
Basic							
Age	< 40	149	11(7.38)	0.465	0.219	0.986	0.042
	> = 40	164	24(14.63)				
Sex	male	226	32(14.16)	4.619	1.376	15.501	0.007
	female	87	3(3.45)				
Health care-related	yes	21	7(33.33)	4.714	1.756	12.654	0.003
	no	292	28(9.59)				
Intravenous drug users	yes	51	10(19.61)	2.312	1.034	5.171	0.037
	no	262	25(9.54)				
Clinical findings							
Hemorrhagic stroke	yes	27	10(37.04)	6.141	2.541	14.840	< 0.001
	no	286	25(8.74)				
Embolism	yes	82	15(18.29)	2.362	1.145	4.870	0.017
	no	231	20(8.66)				
Ischemic stroke	yes	69	14(20.29)	2.703	1.292	5.653	0.007
	no	244	21(8.61)				
Heart insufficiency	yes	182	27(14.84)	2.678	1.175	6.103	0.016
	no	131	8(6.11)				
Acute congestive heart failure	yes	67	21(31.34)	7.565	3.586	15.961	< 0.001
	no	246	14(5.69)				
Renal insufficiency	yes	28	12(42.86)	8.543	3.610	20.217	< 0.001
	no	285	23(8.07)				
Pneumonia	yes	145	25(17.24)	3.292	1.523	7.115	0.002
	no	168	10(5.95)				
Pleural effusion	yes	135	21(15.56)	2.158	1.053	4.421	0.033
	no	178	14(7.87)				
Albumin	< 30 g/L	147	24(16.33)	2.747	1.297	5.848	0.007
	> 30 g/L	166	11(6.63)				
Microorganism							
Blood culture	Positive	181	25(13.81)	1.955	0.905	4.225	0.084
	Negative	132	10(7.58)				
*Staphylococcus aureus*	yes	46	9(19.57)	2.255	0.980	5.188	0.051
	no	267	26(9.74)				
Fungi	yes	6	3(50.00)	8.594	1.664	44.375	0.002
	no	307	32(10.42)				
Echocardiography							
Vegetaion	Negative	39	0(0.00)	1.146	1.096	1.200	0.036
	Positive	181	35(19.34)				
Left heart	yes	199	28(14.07)	2.503	1.056	5.931	0.032
	no	114	7(6.14)				
Both left and right heart	yes	9	4(44.44)	7.045	1.797	27.621	0.007
	no	304	31(10.20)				
Valve type	Prosthetic	11	4(36.36)	4.995	1.384	18.029	0.027
	Native	302	31(10.26)				
Surgery treatment	yes	187	9(4.81)	0.194	0.088	0.431	< 0.001
	no	126	26(20.63)				
Early sugery	yes	160	6(3.75)	0.167	0.067	0.414	< 0.001
	no	153	29(18.95)				

*P* value were estimated by Chi-squared tests or †Fisher exact tests

**Table 7:** Multivariate predictors of in-hospital mortality

Factor	B	OR	95% CI		*P*
			lower	upper	
Intravenous drug users	1.456	4.290	1.098	16.758	0.036
Prosthetic valve endocarditis	1.998	7.374	1.177	46.179	0.033
Hemorrhagic stroke	1.759	5.804	1.830	18.413	0.003
Acute congestive heart failure	2.362	10.607	3.842	29.284	< 0.001
Renal insufficiency	2.227	9.268	2.924	29.382	< 0.001
Left-sided endocarditis	1.724	5.606	1.461	21.512	0.012
Early surgery	−2.311	0.099	0.030	0.330	< 0.001
Constant	−13.894				< 0.001

The goodness-of-fit of the multivariable model was determined by Hosmer–Lemeshow test (Chi-square = 1.562, *P* = 0.955)

## Discussion

IE is a fatal disease with diversity of clinical manifestations and risk factors, continuing to be associated with high mortality despite of novel diagnostic and therapeutic strategies [1]. The demographics, predisposing factors, clinical features, and microbiological spectrum of IE have evolved in recent decades. Relative studies remain scarce in China, and are usually of small sample. Our study was aimed to better understand the regional characteristics and the changing profile of IE over 18 years in our hospital, and to evaluate independent factors that influence the outcome of IE. To our knowledge, this is the largest study on IE performed in our region over 18 years.

### Clinical features

Many studies detected an increase in cases of IVDU-related IE, a trend that has been documented in Australia [15], America [16–18] and Sweden [19]. Conversely, in our study, the proportion of IVDU-related IE declined by half in later-period group as the Chinese government had been stepping up efforts to crack down drug cartels [20], which might play an important reason for the changing profile of IE for 18 years in our region. IE patients in developed countries [8, 21–24] were markedly older than developing regions [10, 24–26]. The mean age of IE patients in the International Collaboration on Endocarditis–Prospective Cohort Study (ICE-PCS), the largest cohort study of IE worldwide, was 57.9 years old [6], far older than ours (42.3 years old). As the young are more likely to be exposed to drugs compared to the middle-age and the old [17, 27], the downward trend of intravenous drugs abusing may be responsible for upward tendency of onset age in the later-period group. It is generally known that IVDU-related IE is more likely to be *Staphylococcus aureus*-related, and usually more frequently occur on tricuspid valve [27, 28]. With the significantly lower proportion of IVDUs, *Staphylococcus aureus* cultured from blood and vegetations on tricuspid valve decreased strikingly in the later-period group. Meanwhile, the decrease of patients with pulmonary embolism in the later-period group could be explained by less numerous right-sided IE. Besides, the lower occurrence of renal insufficiency in the later-period group might benefit from the reduction in *Staphylococcus aureus,* which was perceived as a risk factors for acute renal failure in some study [29].

Beyond the IVDU-related IE, there were still some other points below worth mentioning.

IE patients of the later-period group developed less ischemic stroke. Previous studies reported that *Staphylococcus aureus* infection and vegetations on the mitral valve were risk factors for ischemic stroke [30, 31], but among the patients in this study, the later-period group showed a lower percentage of *Staphylococcus aureus* infection and a nonsignificant rise in patients with mitral vegetations. We speculate that an older age at onset and a higher proportion of diabetics may play a more important role in triggering ischemic stroke.

The ICE-PCS reported that 87.1% of cases had echocardiographic evidence of vegetation [6], similar to our data. The negative echocardiography results (absence of vegetations) is still a stumbling block to diagnosis, which increased significantly in the later-period group. The most frequent explanations for a negative echocardiogram are very small vegetations, non-oscillating and/or atypically located vegetations, or severe, pre-existing lesions from rheumatic heart disease or degenerative heart disease in heart valves [32]. For suspected cases or cases with negative TTE, especially when a prosthetic heart valve or an intracardiac device is present, the appliance of TOE is strongly recommended [11, 32]. However, we observed that TOE was rarely applied to above cases in our study, which exactly need an improvement.

Up to 41.8% of patients were blood-culture negative in our study, which was similar to other region of China (from 31.4 to 51%) [10, 26, 33]. According to the available literature, the incidence of BCNE has been reported to be 7% in North America [6], 5.2–24% in Europe [9, 24, 34, 35], 20% in Japan [36], 20% in South America [6], 31–69% in South Asia [24, 37, 38]. Therefore we could draw a conclusion that BCNE occurs more frequently in developing countries. BCNE is associated with inappropriate antibiotic treatment, faulty culture techniques, atypical pathogens that are difficult to culture or identify [39]. Among these factors, the misuse and overuse of antibiotics remained a problem, especially for patients with long-term fever. Atypical pathogens can be identified by serological analysis and polymerase chain reaction (PCR) assays of blood and pathological specimens [40], which is difficult to realize in clinical practice due to economic and subjective factors. With the development of improved microbial culture techniques, increased medical expertise, and more accurate specifications for the diagnostic and treatment processes, the negative blood-culture rate achieved a remarkable decline in the later-period group. Still, there is room for improvement and research efforts need to be continued.

A systematic review of 21 regional literatures in the world revealed that the average fatality rate of IE is 21.1% ± 10.4% [2], and the ICE-PCS pointed out the in-hospital mortality was 18% worldwide by average [6]. The in-hospital mortality of our study was 11.2%, nearly approaching to the lower limit and quite similar to another research conducted in East China (10.9%). Moreover, it is noteworthy that even with the novel diagnostic and therapeutic strategies available now, the in-hospital mortality did not strikingly differ between the two groups, which means minimizing the in-hospital mortality of IE is still a long-term undertaking.

### Risk factors for in-hospital mortality

To explore the independent risk factors for in-hospital mortality, we performed a forward stepwise logistic regression analysis model. The results indicated that IVDUs, prosthetic valve endocarditis [6, 41], hemorrhagic stroke, acute congestive heart failure [26, 42–44], renal insufficiency [42], left-sided endocarditis and early surgical treatment [6, 44–46] were the independent determinants of in-hospital mortality. Among these factors, prosthetic valve endocarditis had the highest odds ratio. Many of them are also confirmed by previous researches. Some factors, such as age, embolism (or Ischemic stroke), health-related endocarditis. Were finally ruled out from forward stepwise method logistic regression analysis model, probably due to the multicollinearity with other variables. In other studies, increasing age, health care-associated IE, *Staphylococcus aureus* related IE, coagulase-negative staphylococcal infection, paravalvular complications and diabetes mellitus [6, 8, 47, 48] are also important factors contributing to the in-hospital mortality. These discrepancies may due to differences in samples and study design.

Early surgery has been proved to be associated with a significantly lower in-hospital mortality rate as compared to medical therapy [49, 50] Mortality of patients who underwent surgery was one sixth of that of patients who did not have the surgery. In our study, up to 59.7% of our patients underwent surgery during hospitalization, which is similar to other regions like Brazil (52.4–55.0%) [43], Spain (57.0%) and France (31.0–71.0%) [34], but relatively higher compared to Japan (17.0%) [8] and North America (45.0%). The ICE-PCSS showed that 46% of patients worldwide underwent early surgery [46]. In our studies, nearly 51.1% of cases were admitted to early surgery, which turned to be the only protective factor for prognosis of IE in our multivariate model. We believe that good standard of care in our hospital, and relatively younger age were a major reason for patients to make aggressive decision of surgical treatment.

The difference of in-hospital mortality between IVDU-related IE and none-IVDU-related IE was reported to be of no significance in previous studies [18, 51, 52], inconsistent with our conclusion. We speculated that the higher *Staphylococcus aureus* septicemia and repeated infection brought by intravenous drugs busing might contribute to the higher in-hospital mortality. We strongly proposed to conduct more further studies so as to verify our conclusions.

### Limitation

This study focused on a single-center in a general teaching hospital without long-term follow-up. Most patients came from south China, thus findings in this study may not be applicable to all populations. Besides, referral bias should be taken into consideration when describing the clinical spectrum and outcome of IE, as patients with more complications such as stroke, heart failure and new valvular regurgitation and surgery indications, who are more likely to be gravely ill patients, are more likely to choose a tertiary hospital [53]. So our conclusions may not apply to small hospital. However, our observations reflected a dynamic change of IE in our center over a period of eighteen consecutive years with a relatively large sample size, while relative study remains scarce in China. The geographic variations observed in our study will be of important value to profile the clinical feature of China and offer the reference for clinical decisions in our region.

## Conclusion

In conclusion, intravenous drug abuse was less common in later-period group, which might result in a series of changes like older age of onset, fewer pulmonary embolism, renal failure, *Staphylococcus aureus* endocarditis and right-sided IE. More ischemic stroke was observed possibly due to older age. Also, patience in later-period showed more splenomegaly, lower BCNE rate and negative echocardiography results. The in-hospital mortality stayed still despite of the changing profile of IE. The multivariate analysis underlined the significance of prosthetic valve endocarditis, intravenous drug addicted, hemorrhagic stroke, congestive heart failure, renal insufficiency, left-sided endocarditis, fungal endocarditis and surgical treatment to in-hospital mortality.

## Data Availability

The datasets used and/or analyzed during the current study are available from the corresponding author on reasonable request.

## Abbreviations

BC: Blood culture
CI: Confidence interval
CT: Computed Tomography
ESC: European Society of Cardiology.
FDG: Fluorodeoxyglucose
IE: Infective endocarditis
IVDU: Intravenous drug users
NCBC: Blood culture-negative infective endocarditis
OR: Odds ratio
PCR: Polymerase chain reaction
TTE: Transthoracic echocardiography
